# Evaluation of optical coherence tomography angiography metrics in children and adolescents with type 1 diabetes: 4-year longitudinal study

**DOI:** 10.1007/s00592-024-02291-4

**Published:** 2024-05-03

**Authors:** Xinran Qin, Ying Xiao, Lipu Cui, Shuli Chen, Qingyu An, Tianyi Yuan, Yiwei Wu, Qiurong Lin, Chenhao Yang, Haidong Zou

**Affiliations:** 1 Department of Ophthalmology, Shanghai General Hospital, Shanghai Jiao Tong University School of Medicine, Shanghai, China; 2 Shanghai Eye Diseases Prevention & Treatment Center, Shanghai Eye Hospital, No.100 Haining Road, Shanghai, 200080 China; 3 Department of Ophthalmology, Children’s Hospital of Fudan University, No. 399 Wanyuan Road, Shanghai, 201102 China; 4 Shanghai Key Laboratory of Fundus Diseases, Shanghai, China; 5 National Clinical Research Center for Eye Diseases, Shanghai, China; 6 Shanghai Engineering Center for Precise Diagnosis and Treatment of Eye Diseases, Shanghai, China

**Keywords:** Type 1 diabetes, Diabetic retinopathy, Optical coherence tomography angiography

## Abstract

**Purpose:**

To evaluate longitudinal changes in optical coherence tomography angiography (OCTA) metrics in children and adolescents with type 1 diabetes (T1D).

**Methods:**

This prospective observational cohort study included thirty-two eyes from thirty T1D children with no history of diabetic retinopathy (DR) who were followed up for 4 years. Participants underwent OCTA examinations at baseline and during follow-up. Quantitative OCTA metrics were measured using a customized MATLAB algorithm. Generalized mixed-effect models were used to determine their relationship with DR development. Systemic parameters and OCTA metrics were screened using least absolute shrinkage and selection operator to identify predictors for visual function.

**Results:**

Over the 4-year period, seven of the included eyes developed DR, and most OCTA metrics decreased with diabetes duration. Higher peripapillary and parafoveal nasal quadrant vessel area density (VAD) in the superficial capillary plexus (SCP) and vessel skeleton density (VSD) in both the SCP and the deep capillary plexus (DCP) were associated with a lower risk of DR in T1D. Parafoveal DCP VSD and VAD in the temporal and inferior quadrants were anticorrelated with changes in best corrected visual acuity.

**Conclusions:**

OCTA metrics dynamically change over the duration of diabetes and can be used as biomarkers to improve the risk evaluation of DR development and visual function in T1D children and adolescents.

**Supplementary Information:**

The online version contains supplementary material available at 10.1007/s00592-024-02291-4.

## Introduction

Type 1 diabetes (T1D) is a chronic health condition characterized by autoimmune destruction of beta cells in the pancreatic islets, resulting in an absolute insulin deficiency. Globally, T1D affects over 8.4 million people, with 1.5 million of them aged younger than 20 years in 2021 [1]. Despite significant progress in the management of T1D, modifying the risk of long-term complications remains a primary concern, especially for pediatric-onset patients. Diabetic retinopathy (DR) is one of the most common complications of T1D and the leading cause of visual impairment [2]. To address this, the International Society for Pediatric and Adolescent Diabetes recommends annual screening for retinopathy by slit lamp or fundus photography, starting at age 11 and for those with 2 to 5 years of diabetes duration [3]. Adding a layer of complexity to the disease prevention paradigm, in vivo studies have suggested that retinal neurodegeneration and microvascular regression may precede clinically detectable retinopathy signs in fundus images [4]. Furthermore, established risk factors (such as diabetes duration and glycated hemoglobin [HbA1c]) have proven insufficient in predicting the development of DR [5].

Advancements in optical coherence tomography angiography (OCTA) technology have enabled noninvasive assessment of retinal microvasculature and capillary perfusion. In addition to offering a three-dimensional structural visualization, OCTA excels in precise quantification of vessel features by tracking red blood cell motion. With the increased availability of OCTA, cumulative studies have established a strong association between DR and OCTA-derived metrics. For example, Tan and colleagues demonstrated the remarkable accuracy of wide-field OCTA (12 × 12-mm field) in grading the severity of nonproliferative DR (NPDR) [6]. Takase et al. suggested that changes in the size and circularity of the foveal avascular zone (FAZ) could be observed before DR development, potentially serving as indicators for early detection of DR [7]. Additionally, a prospective study by Cheung et al. reported that the FAZ area, vessel density, and fractal dimension of the deep capillary plexus (DCP) can predict DR progression [8]. These findings provide robust support for the application of OCTA in evaluating DR risk.

There is imperative need for longitudinal evidence demonstrating the capacity of quantifiable OCTA metrics to reveal invaluable insights into vascular health and disease in juvenile T1D patients. In this study, we leveraged a prospective observational study to unravel the alterations in retinal vasculature characteristics among T1D children and adolescents over a span of 4 years. We also explored the possible correlation of OCTA metrics with systemic factors, delving deeper into their predictive capacity for the onset of DR and vision loss. Our study offers a novel and longitudinal perspective on retinal imaging traits as prognostic factors, shedding light on early microvascular abnormalities in the retina and their role in predicting visual impairment in pediatric patients with T1D.

## Methods

### Participants

This 4-year longitudinal cohort study was conducted as a part of the *SCADE* project (Shanghai Children and Adolescent Diabetes Eye study, ClinicalTrials.gov identifier: NCT 03587948) [9]. In brief, the *SCADE* is a prospective cohort study spanning from January 2018 to 2028. The overarching aim of this project is to recruit 300 children with T1D in Children's Hospital of Fudan University in Shanghai as the case group, along with 300 healthy children without diabetes as the control group. To guide disease screening of children and adolescents with diabetes is the primary determination of the *SCADE* study. It also seeks for providing comprehensive, continuous, and dynamic information to diabetic eye disease management.

A total of 30 children and adolescents who finished the year-four visit were included in this longitudinal study, additional 64 individuals who participated in the 2023 visit were designated for the cross-sectional validation study. Main inclusion criteria were (1) age younger than 20 years, in accordance with the World Health Organization’s definition of adolescence [10]; (2) a confirmed diagnosis of T1D following the American Diabetes Association criteria [11]; and (3) a compliance with all examinations at baseline and during follow-up. Those who met any of the following criteria were excluded: (1) the presence of eye diseases that may interfere with OCTA examinations, such as inherited retinopathy, high myopia, keratitis, scleritis, and cataracts; (2) a history of systemic diseases other than T1D, such as hypertension and systemic lupus erythematosus; and (3) eyes with recent intraocular surgeries, laser treatment or intravitreal therapy within the preceding 6 months.

The present study adhered to the tenets of the Declaration of Helsinki and was approved by the Ethics Committee of the Shanghai General Hospital of Shanghai Jiao Tong University and the Children's Hospital of Fudan University in Shanghai (approval number, 2016KY005 and LS No. 01 (2018)). All participants provided written informed consent.

### Data collection

At baseline and follow-up visits, parents of all participants completed a questionnaire providing demographic information, including sex, birth date, history of T1D and diabetic complications. Anthropometric measurements (height, weight, and blood pressure) were recorded for all subjects, and ophthalmic examinations were conducted by trained examiners using standardized protocols and instruments. These examinations included (1) slit lamp examination (SL130; Zeiss, Germany), (2) digital nonmydriatic fundus photography (AFC-210; NIDEK, Tokyo, Japan), (3) intraocular pressure (IOP) measurement (NT-530P; NIDEK), (4) IOL Master 700 assessments (Carl Zeiss Meditec, Dublin, CA, USA), (5) best-corrected visual acuity (BCVA) measured using the international standard LogMAR visual acuity chart after adequate pupil dilation (1% tropicamide, 3 drops in total, one at a time, at 5-min intervals) [12], (6) refractive power measured by optometry (KR-8900; Topcon, Tokyo, Japan), and (7) OCTA (CIRRUS HD-OCT Model 5000; Carl Zeiss Meditec) scanning a 6 × 6-mm acquisition protocol centered at the macula and the optic disc in sequence [13]. To ensure the quality of the OCTA examinations, eye motion artifacts were minimized by the CIRRUS eye-tracking algorithm during image acquisition. Poor-quality images (defined as a signal strength index < 6/10) were retaken immediately unless subjects were unable to cooperate with continued examination.

For the definition of DR development, retinal fundus photographs were taken using a nonmydriatic camera after pharmacologic pupil dilation. Two photographs of each eye were taken at each visit, one centered at the optic disc and the other centered at the fovea. Both baseline and follow-up photographs were assessed using the Early Treatment of Diabetic Retinopathy Study severity grading scale by the masked grader.

### OCTA image analysis

The original OCTA images were automatically segmented by the built-in software into the superficial capillary plexus (SCP) and the DCP. The examiner carefully scrolled the B-scans to maintain accuracy and eliminate major errors. Following this, quality control was carried out by masked graders in a standardized environment. Images with significant artifacts, such as blur, defocus, extensive eye motion, and fixation problems, were excluded from subsequent analysis (N = 6). In our quantitative OCTA analysis, a customized MATLAB algorithm (The MathWorks Inc), as previously detailed by Wang et al. [14], was used to yield measurements. The reliability and repeatability of the measurements have been reported by Zhao and colleagues [15]. The following metrics were computed: (1) vessel area density (VAD), which quantifies the total area of perfused vasculature per unit area in a region of measurement; (2) vessel skeleton density (VSD), which denotes the total length of vessels containing particles with velocities ranging between 0.3 and 3 mm/sec; (3) FAZ size. Additionally, VAD and VSD were assessed in four quadrants surrounding either the macular fovea or optic disc. To adjust the ocular magnification from axial eye growth, we manually calculated scaling factors for these metrics using a previously described formula [16–18].

### Statistical analysis

Data distribution was examined using the Shapiro–Wilk test. Demographic and clinical characteristics were compared between baseline and follow-up using a paired t test (or Wilcoxon rank-paired test when appropriate) for continuous variables and Fisher’s exact test for categorical variables. In addressing the inter-eye correlation inherent in observations made on the same patient when comparing eye-level characteristics, a generalized estimating equation (GEE) model was employed. To test for differences in the OCTA metrics between baseline and follow-up, paired t test followed by false discovery rate correction was used. Subsequently, linear regression analyses with a GEE framework were performed to identify systemic determinants underlying the longitudinal changes in OCTA metrics.

A generalized mixed-effect regression model was used to determine the association between OCTA metrics and DR development. Random effects included the subject ID alone or in combination with known influential factors, while individual OCTA metrics were assessed as fixed effects to discern their performance in distinguishing control eyes from eyes with NPDR. The predictive value was quantified by calculating the area under the receiver operator characteristic curve (AUC). To validate the discrimination of selected variables, Firth’s bias-reduced logistics regression was used in the validation cohort. For evaluating the association of OCTA metrics with the time to DR development, Firth’s bias-reduced Cox proportional hazards model was used. An unadjusted Cox regression model was first constructed, followed by a minimally adjusted model that included sex, duration of diabetes at baseline, and current HbA1c.

The influence of parafoveal metrics on visual function, as indicated by the changes in BCVA, was analyzed. Least absolute shrinkage and selection operator (LASSO) regression was used to select the most predictive variables that were determined by the minimum λ. Afterward, factors identified by LASSO analysis were individually set as covariate of the GEE linear regression model with BCVA as dependent variable. Goodness of fit was measured using the adjusted R-squared values.

All statistical analyses were performed using R 4.2.3 (R Foundation for Statistical Computing, Vienna, Austria) with statistical significance set at a two-tailed *P*-value < 0.05. For multiple comparisons, Benjamini–Hochberg method was used to correct false discovery rate with significance set at q < 0.05.

## Results

### Participant characteristics

This longitudinal study included 32 eyes from 30 patients who underwent a minimum of two follow-ups over a 4-year period. An overview of the demographic, systemic, and ophthalmic characteristics of the participants both at baseline and year-four follow-up was summarized in Table 1. At baseline, participants had a mean age of 11.1 years, with 19 of them being female. Their average duration of T1D was 3.75 ± 2.46 years, and their initial LogMAR BCVA was 0.017 ± 0.065. Over 4 years, most parameters significantly differed from baseline, including increases in height, weight, body mass index (BMI) and axial length (AL), as well as notable decreases in diastolic blood pressure (DBP), spherical equivalent (SE) refraction, and visual acuity (VA).

**Table 1:** Demographic and clinical characteristics of study population

	Baseline (*n* = *30*)	Follow-up (*n* = *30*)	p-value
*Demographics*			
Age, y	11.07 ± 3.33	15.07 ± 3.33	/
Female, %	19 (63.3%)	19 (63.3%)	/
Height, cm	143.0 ± 17.2	159.2 ± 10.3	**< 0.001**^**b**^
Weight, kg	40.1 ± 12.87	54.1 ± 11.7	**< 0.001**^**a**^
BMI, kg/m^2^	19.02 ± 2.37	21.13 ± 2.91	**< 0.001**^**a**^
Duration of diabetes, y	3.75 ± 2.46	7.75 ± 2.46	/
SBP, mmHg	110.6 ± 17.45	117.6 ± 11.1	0.219^a^
DBP, mmHg	74.19 ± 16.22	67.36 ± 8.97	**0.013**^**a**^
AL, cm	23.71 ± 1.17	24.34 ± 1.20	**0.003**^**b**^
SE, D	− 1.20 ± 2.41	− 2.70 ± 2.57	**< 0.001**^**b**^
VA, LogMAR	0.100 ± 0.18	0.356 ± 0.311	**< 0.001**^**b**^
BCVA, LogMAR	− 0.017 ± 0.065	− 0.012 ± 0.061	0.625^b^
IOP, mmHg	16.67 ± 2.79	15.23 ± 2.90	0.053^a^
HbA1_c_, %	7.45 ± 1.88	7.50 ± 1.22	0.779^a^
*Severity of retinopathy*			**0.011**^**c**^
No DR, %	30 (100%)	23 (76.7%)	/
NPDR, %	0 (0%)	7 (23.3%)	/

*AL* axial length, *BCVA* best corrected visual acuity, *BMI* body mass index, *DBP* diastolic blood pressure, *DR* diabetic retinopathy, *HbA1c* glycemic hemoglobin, *IOP* intraocular pressure, *NPDR* non-proliferative diabetic retinopathy, *SBP* systolic blood pressure, *SE* spherical equivalent, *VA* visual acuity

^a^Paired t test; ^b^Wilcoxon matched-pairs signed rank test; ^c^Fisher’s exact test

During the follow-up period, 7 of 32 (22%) eyes without a retinopathy history at baseline developed NPDR according to the lesions present in their fundus images. Notably, as current therapies for Diabetic Retinopathy (DR) primarily target advanced stages, none of these individuals received additional treatment beyond regular insulin therapy. Despite these alterations, there was no significant change in the mean BCVA (− 0.017 ± 0.065 LogMAR at baseline versus − 0.012 ± 0.061 LogMAR at follow-up, *P* = 0.625). For the validation cohort, 11 out of 64 eyes were diagnosed with NPDR. The DR group and the control were matched for all demographic and clinical characteristics (Supplementary Table 1).

### Longitudinal changes in OCTA metrics in children with T1D

**Fig. 1:**
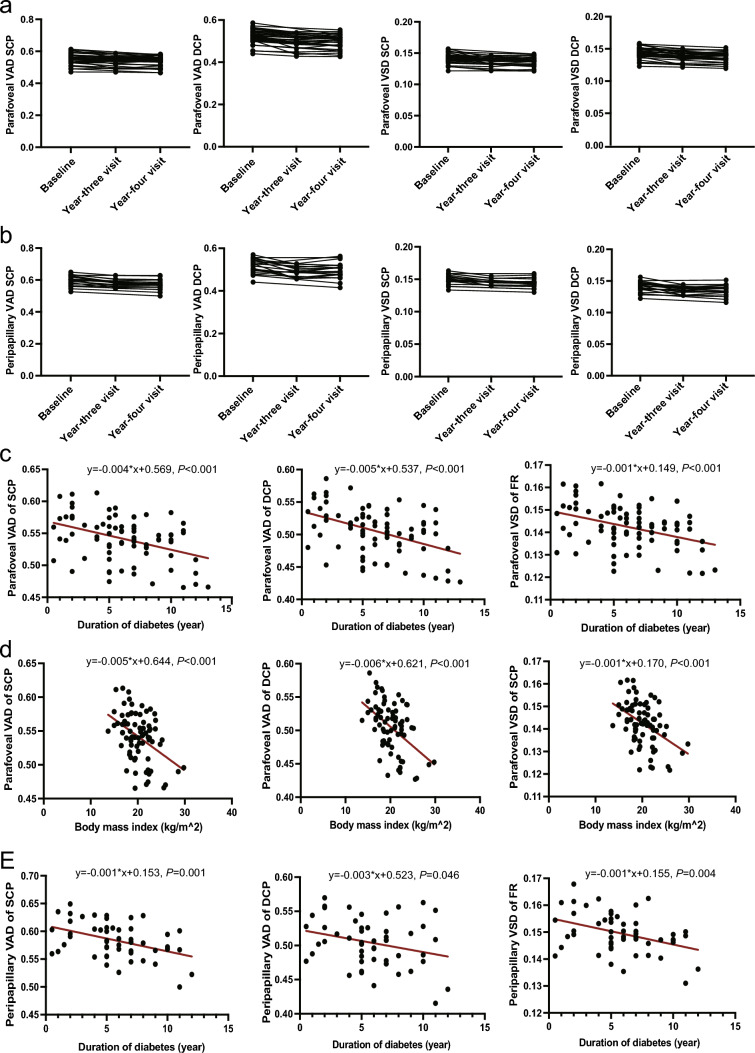
Longitudinal changes in OCTA metrics during follow-up and influential factors. **a** line charts of parafoveal vessel area density (VAD)/vessel skeleton density (VSD) in the superficial capillary plexus (SCP) and deep capillary plexus (DCP) at baseline, year-three visit and year-four visit; **b** line charts of peripapillary VAD /VSD in the SCP and DCP at baseline, year-three visit and year-four visit; **c** scatterplots showing VAD in the SCP and DCP and VSD of the full retina in linear (red line) and regression models with diabetes duration; **d** scatterplots showing VAD in the SCP and DCP and VSD of the full retina in linear (red line) and regression models with body mass index

Of the 32 eyes included, a substantial number of AL-corrected metrics at the year-four follow-up visit showed significant reductions compared to baseline (Fig. 1a and b, Table 2). Notably, the absolute decline in peripapillary metrics is more pronounced in the SCP as opposed to the DCP, such as total VAD (SCP: 0.596 ± 0.033 *versus* 0.571 ± 0.031; DCP: 0.517 ± 0.034 *versus* 0.497 ± 0.037; paired t test; all *P* < 0.001, q < 0.05). Conversely, the macular metrics displayed a more pronounced decline in the DCP than the SCP, including total VAD (SCP: 0.553 ± 0.035 *versus* 0.536 ± 0.033; DCP: 0.520 ± 0.035 *versus* 0.498 ± 0.033; paired t test; all *P* < 0.001, q < 0.05) and total VSD (SCP: 0.142 ± 0.008 *versus* 0.138 ± 0.007; DCP: 0.144 ± 0.009 *versus* 0.139 ± 0.008; paired t test; all *P* < 0.001, q < 0.05). Furthermore, quadrant analysis revealed that perfusion reduction in different areas were inconsistent (Table 2). Specifically, certain areas such as parafoveal VAD in the superior and inferior quadrants of the DCP, and superior quadrant VAD and VSD of the peripapillary DCP showed no significant changes over the 4 years. In contrast, considerable decreases in VAD and VSD of the temporal and nasal quadrants were observed in all segments of the parafoveal and peripapillary regions, suggesting their vulnerability to diabetes-related perfusion deficits.

**Table 2:** Comparison of the OCTA-metrics between the eyes at baseline and 4-year follow-up

	Baseline				Year Four Follow-up					
Parafoveal (N = 32 pairs)	Mean	Std. Deviation	Lower 95% CI	Upper 95% CI	Mean	Std. Deviation	Lower 95% CI	Upper 95% CI	*P*-value	*Q-value*
Parafoveal VAD of FR	0.558	0.039	0.544	0.573	0.540	0.037	0.527	0.554	**< **0.001	**< **0.001
Parafoveal VSD of FR	0.146	0.010	0.142	0.149	0.141	0.008	0.138	0.144	**< **0.001	**< **0.001
Parafoveal VAD of SCP	0.553	0.035	0.541	0.566	0.536	0.033	0.524	0.547	**< **0.001	**< **0.001
Parafoveal VSD of SCP	0.142	0.008	0.139	0.145	0.138	0.007	0.135	0.140	**< **0.001	**< **0.001
Parafoveal VAD of DCP	0.520	0.035	0.508	0.533	0.498	0.033	0.486	0.510	**< **0.001	**< **0.001
Parafoveal VSD of DCP	0.144	0.009	0.141	0.147	0.138	0.008	0.135	0.141	**< **0.001	**< **0.001
Parafoveal VAD temporal quadrant of FR	0.577	0.044	0.561	0.593	0.559	0.043	0.544	0.575	0.001	0.002
Parafoveal VAD superior quadrant of FR	0.568	0.044	0.552	0.583	0.553	0.040	0.539	0.568	0.019	0.023
Parafoveal VAD nasal quadrant of FR	0.541	0.044	0.525	0.557	0.524	0.054	0.505	0.544	0.057	0.063
Parafoveal VAD inferior quadrant of FR	0.552	0.050	0.534	0.570	0.537	0.045	0.521	0.554	0.017	0.021
Parafoveal VSD temporal quadrant of FR	0.153	0.011	0.149	0.157	0.148	0.010	0.144	0.152	**< **0.001	**< **0.001
Parafoveal VSD superior quadrant of FR	0.149	0.011	0.145	0.153	0.146	0.010	0.143	0.150	0.013	0.018
Parafoveal VSD nasal quadrant of FR	0.145	0.010	0.141	0.149	0.141	0.012	0.136	0.145	0.014	0.019
Parafoveal VSD inferior quadrant of FR	0.146	0.013	0.141	0.150	0.141	0.011	0.138	0.145	0.005	0.009
Parafoveal VAD temporal quadrant of SCP	0.568	0.041	0.553	0.583	0.551	0.041	0.536	0.566	**< **0.001	**< **0.001
Parafoveal VAD superior quadrant of SCP	0.562	0.040	0.548	0.576	0.544	0.037	0.531	0.557	0.002	0.004
Parafoveal VAD nasal quadrant of SCP	0.530	0.041	0.515	0.544	0.511	0.048	0.494	0.529	0.017	0.021
Parafoveal VAD inferior quadrant of SCP	0.547	0.046	0.531	0.564	0.532	0.041	0.517	0.547	0.005	0.009
Parafoveal VSD temporal quadrant of SCP	0.148	0.010	0.144	0.152	0.144	0.010	0.140	0.147	**< **0.001	**< **0.001
Parafoveal VSD superior quadrant of SCP	0.145	0.010	0.141	0.148	0.141	0.008	0.138	0.144	0.001	0.002
Parafoveal VSD nasal quadrant of SCP	0.139	0.009	0.136	0.142	0.135	0.010	0.131	0.139	0.004	0.008
Parafoveal VSD inferior quadrant of SCP	0.141	0.011	0.137	0.145	0.137	0.009	0.134	0.141	0.001	0.002
Parafoveal VAD temporal quadrant of DCP	0.539	0.043	0.523	0.555	0.519	0.048	0.502	0.536	0.006	0.009
Parafoveal VAD superior quadrant of DCP	0.532	0.041	0.517	0.546	0.516	0.052	0.498	0.535	0.059	0.063
Parafoveal VAD nasal quadrant of DCP	0.523	0.035	0.511	0.536	0.508	0.046	0.492	0.525	0.046	0.053
Parafoveal VAD inferior quadrant of DCP	0.513	0.047	0.496	0.530	0.502	0.051	0.483	0.520	0.105	0.105
Parafoveal VSD temporal quadrant of DCP	0.150	0.010	0.146	0.153	0.144	0.010	0.141	0.148	**< **0.001	**< **0.001
Parafoveal VSD superior quadrant of DCP	0.149	0.010	0.146	0.153	0.144	0.012	0.140	0.148	0.001	0.002
Parafoveal VSD nasal quadrant of DCP	0.144	0.009	0.141	0.147	0.140	0.010	0.136	0.143	0.006	0.009
Parafoveal VSD inferior quadrant of DCP	0.144	0.012	0.140	0.149	0.141	0.011	0.137	0.145	0.008	0.012
FAZ size	0.338	0.109	0.299	0.377	0.320	0.130	0.273	0.366	0.085	0.088
*Peripapillary (N* = *21 pairs)*										
Peripapillary VAD of FR	0.599	0.034	0.584	0.615	0.575	0.032	0.561	0.590	**< **0.001	**< **0.001
Peripapillary VSD of FR	0.152	0.008	0.149	0.156	0.147	0.007	0.144	0.151	**< **0.001	**< **0.001
Peripapillary VAD of SCP	0.596	0.033	0.581	0.610	0.571	0.031	0.557	0.585	**< **0.001	**< **0.001
Peripapillary VSD of SCP	0.149	0.007	0.146	0.153	0.144	0.007	0.141	0.147	**< **0.001	**< **0.001
Peripapillary VAD of DCP	0.518	0.034	0.502	0.533	0.497	0.037	0.480	0.514	0.002	0.003
Peripapillary VSD of DCP	0.141	0.008	0.137	0.145	0.135	0.008	0.132	0.139	**< **0.001	**< **0.001
Peripapillary VAD temporal quadrant of FR	0.624	0.037	0.607	0.641	0.598	0.039	0.580	0.615	**< **0.001	**< **0.001
Peripapillary VAD superior quadrant of FR	0.670	0.032	0.655	0.684	0.651	0.035	0.635	0.667	0.003	0.004
Peripapillary VAD nasal quadrant of FR	0.620	0.037	0.603	0.637	0.581	0.039	0.563	0.598	**< **0.001	**< **0.001
Peripapillary VAD inferior quadrant of FR	0.660	0.038	0.643	0.678	0.636	0.038	0.618	0.653	0.001	0.002
Peripapillary VSD temporal quadrant of FR	0.159	0.008	0.155	0.162	0.152	0.008	0.149	0.156	**< **0.001	**< **0.001
Peripapillary VSD superior quadrant of FR	0.170	0.008	0.167	0.174	0.166	0.008	0.162	0.169	0.001	0.002
Peripapillary VSD nasal quadrant of FR	0.160	0.008	0.156	0.163	0.150	0.008	0.146	0.154	**< **0.001	**< **0.001
Peripapillary VSD inferior quadrant of FR	0.168	0.010	0.163	0.172	0.162	0.009	0.158	0.166	0.001	0.002
Peripapillary VAD temporal quadrant of SCP	0.620	0.0362	0.604	0.637	0.595	0.037	0.578	0.612	0.001	0.002
Peripapillary VAD superior quadrant of SCP	0.671	0.0319	0.656	0.685	0.651	0.035	0.635	0.667	0.001	0.002
Peripapillary VAD nasal quadrant of SCP	0.615	0.0377	0.598	0.632	0.576	0.039	0.558	0.593	**< **0.001	**< **0.001
Peripapillary VAD inferior quadrant of SCP	0.659	0.0373	0.642	0.676	0.635	0.037	0.618	0.652	**< **0.001	**< **0.001
Peripapillary VSD temporal quadrant of SCP	0.155	0.0076	0.152	0.159	0.150	0.008	0.146	0.154	**< **0.001	**< **0.001
Peripapillary VSD superior quadrant of SCP	0.170	0.0082	0.166	0.173	0.164	0.008	0.161	0.168	**< **0.001	**< **0.001
Peripapillary VSD nasal quadrant of SCP	0.157	0.0075	0.154	0.160	0.148	0.009	0.144	0.152	**< **0.001	**< **0.001
Peripapillary VSD inferior quadrant of SCP	0.167	0.0093	0.162	0.171	0.161	0.009	0.157	0.166	**< **0.001	**< **0.001
Peripapillary VAD temporal quadrant of DCP	0.557	0.0329	0.542	0.572	0.535	0.039	0.517	0.553	0.006	0.007
Peripapillary VAD superior quadrant of DCP	0.541	0.0413	0.522	0.559	0.536	0.046	0.515	0.557	0.609	0.609
Peripapillary VAD nasal quadrant of DCP	0.517	0.0408	0.499	0.536	0.482	0.049	0.459	0.504	0.004	0.005
Peripapillary VAD inferior quadrant of DCP	0.547	0.0394	0.529	0.565	0.530	0.049	0.507	0.552	0.038	0.041
Peripapillary VSD temporal quadrant of DCP	0.151	0.0073	0.147	0.154	0.145	0.008	0.141	0.149	**< **0.001	**< **0.001
Peripapillary VSD superior quadrant of DCP	0.149	0.0093	0.145	0.153	0.147	0.010	0.142	0.151	0.179	0.185
Peripapillary VSD nasal quadrant of DCP	0.144	0.0110	0.139	0.149	0.134	0.012	0.129	0.140	**< **0.001	**< **0.001
Peripapillary VSD inferior quadrant of DCP	0.148	0.0087	0.144	0.151	0.144	0.011	0.139	0.148	0.016	0.018

*CI* confidence interval, *DCP* deep capillary plexus, *FAZ* foveal avascular zone, *FR* full retina, *SCP* superficial capillary plexus, *VAD* vessel area density, *VSD* vessel skeleton density; Paired t test;

To gain deeper insights into the predictors underlying these longitudinal changes, we evaluated the associations between OCTA metrics and systemic characteristics using GEE linear regression models. Our results revealed significant inverse correlations between parafoveal metrics and diabetes duration and BMI, whereas peripapillary metrics appeared less influenced by BMI. (Fig. 1c and d; Supplementary Table 1). It’s worth noting that age was identified as a significant predictor for most OCTA metrics, although it explained a relatively small proportion of the variation (R-square < 0.1). Lastly, our results indicated no significant sex-related differences in retinal perfusion and vessel density, and HbA1c levels did not demonstrate a noteworthy influence on VAD and VSD (Supplementary Table 2).

### Association of OCTA metrics with DR development and visual function

**Fig. 2:**
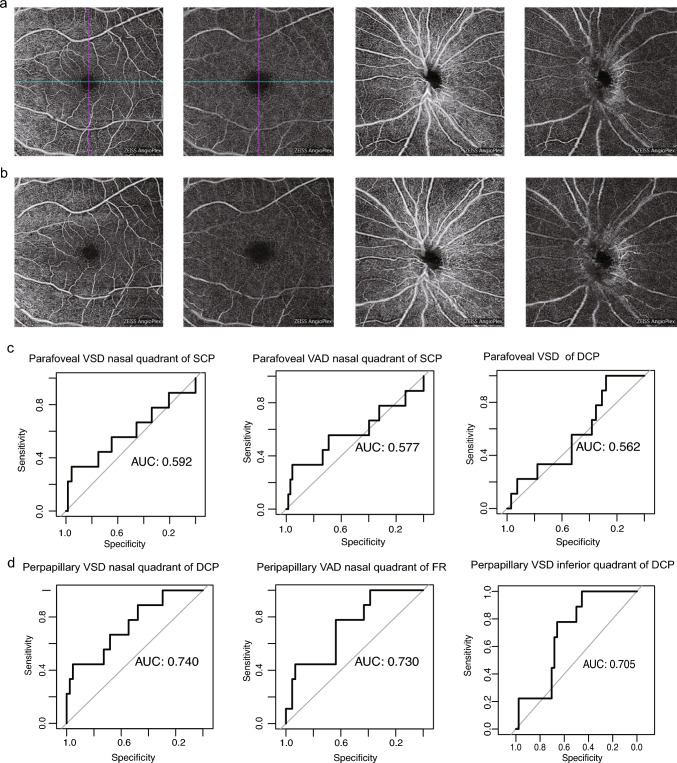
Relationship of OCTA metrics and the development of DR. **a** OCTA en face images of the parafoveal and peripapillary SCP and DCP of a 12-year-old girl who was free of diabetic retinopathy (DR) at baseline; **b** OCTA en face images of the parafoveal and peripapillary SCP and DCP of the same girl who developed nonproliferative DR at year-four visit; **c** receiver operator characteristic (ROC) curves of the most predictive parafoveal metrics for DR risk; **d** ROC curves of the top most predictive peripapillary metrics with corresponding AUCs

During the 4-year follow-up, we observed the development of NPDR in three eyes in 2021 and four more in 2023. This prompted an investigation into the potential associations between changes in OCTA metrics the onset of DR in children and adolescents with T1D. We performed generalized mixed-effect models to explore associations between individual OCTA metrics (considered as fixed effects) and DR occurrence (the dependent variable). To adjust for influential factors, diabetes duration was set as either a random effect or a fixed effect in two separate models, respectively. Among the parafoveal metrics, we identified lower VAD nasal quadrant of the SCP, VSD nasal quadrant of the SCP, and DCP VSD as predictive factors for the development of DR (Fig. 2c; Supplementary Table 3). However, their predictive value was relatively limited (AUC = 0.577, 0.592, 0.565; respectively), and none of them excelled the traditional risk factor duration of T1D alone in predicting DR risk (Bootstrap test, *P* > 0.05). In contrast, decreases in peripapillary metrics showed significant associations with DR occurrence (Fig. 2d; Supplementary Table 3). The most predictive metrics were VSD nasal quadrant of the DCP, VAD nasal quadrant of the FR, and VSD inferior quadrant of the DCP (AUC = 0.740, 0.730, 0.705; respectively). Notably, the discriminatory value of VSD inferior quadrant of the DCP was demonstrated in our validation cohort containing sixty-four eyes (multivariate model adjusted for duration of diabetes, odds ratio: 2.24e-34; 95% confidence interval: 1.02e-72, 0.28; *P* = 0.046). Lastly, we explored time-to-event relationships but found no meaningful correlations in the Firth's bias-reduced Cox regression models. (Supplementary Table 4).

To establish additional structure-to-function connections, we delved deeper into the relationship between parafoveal metrics with visual function as indicated by BCVA. A total of forty parameters, including systemic, ocular, and OCTA-derived, were included in the LASSO model to identify variables with the strongest predictive power. Following LASSO analysis, seven variables were selected as significant predictors of BCVA. These included AL, BMI, HbA1c, VSD of the DCP, VSD in the nasal quadrant of the FR, and VAD in the temporal and inferior quadrants of the DCP. Subsequently, we integrated these LASSO-selected parameters into a GEE linear regression model to further evaluate their predictive value. As a result, AL, BMI, and three OCTA parameters, VSD of the DCP; VAD in the temporal and inferior quadrants of the DCP, maintained their significant associations with changes in BCVA within the final model. (coefficient estimate = 0.005, 0.017, − 1.675, − 0.293, and − 0.232; adjusted R-squared = 0.080, 0.121, 0.100, 0.096, and 0.078; all *P* < 0.05; Supplementary Table 5).

## Discussion

Existing cross-sectional and prospective studies have primarily centered their attention on assessing the predictive utility of macular OCTA metrics in adults with type 2 diabetes [6, 8]. To our knowledge, this longitudinal cohort study stands as the initial attempt to elucidate alterations in retinal vasculature in T1D children and adolescents over a span of 4 years. Additionally, we endeavor to unravel the intricate interrelationship between OCTA metrics, DR occurrence, and visual function. In general, most of the AL-corrected metrics showed a significant decrease during the 4-year period, apart from the FAZ size. It is noteworthy that individuals with an extended duration of diabetes exhibited reduced VAD and VSD in both the macular and disc regions. Eyes with higher VAD and VSD in the nasal quadrant of the retina appeared to have a lower risk for developing DR. We also explored associations between parafoveal metrics and changes in BCVA, pinpointing macular VSD of the DCP, VAD in the temporal and inferior quadrants of the DCP as robust indicators of vision problems in T1D children and adolescents. Ultimately, this study underscores the importance of appropriately adjusting ocular magnification to detect longitudinal variations in OCTA metrics, particularly in adolescent populations undergoing consecutive axial eye growth.

Our findings indicated that the decrease in parafoveal metrics were more pronounced in the DCP during the 4-year period, whereas a more substantial decline in peripapillary metrics were evident in the SCP (Table 2). Histologically, the retinal vascular system is categorized into four distinct strata: (1) radial peripapillary capillaries encircling the optic nerve within the retinal nerve fiber layer (RNFL), (2) SCP located at the retinal ganglion cell layer (RGCL) and the superficial segment of the inner plexiform layer, (3) intermediate capillary plexuses (ICP) situated at the inner boundary of the inner plexiform layer and the superficial portion of the inner nuclear layer, and (4) DCP positioned at the outer border of the inner nuclear layer. The fovea of the macula is devoid of the RNFL, RGCL and inner plexiform layer corresponding to the SCP, and the thickness of the RNFL increases as it approaches the optic disc [19, 20]. These anatomical features explain to some extent the disparities in perfusion defect segmentation between images centered on the macula and the optic disc. Furthermore, OCTA metrics in different quadrants demonstrated varying degrees of change. Specifically, we identified a notable decline in the temporal and nasal quadrants of VAD and VSD over 4 years, in contrast to the relatively stable values observed in the superior and inferior quadrants (Table 2). A prior study by our colleagues unveiled longitudinal alternations in retinal thickness among T1D children and adolescents [21], indicating a reduction in peripapillary RGCL thickness in the temporal sector. Additionally, Cao et al*.* reported that peripapillary RNFL thinning was significant in the nasal quadrant of the retina in preclinical DR patients compared to normal controls [22]. It is thus reasonable to posit that the variations in vessel density across quadrants are inherently linked to structural changes in RNFL and RGCL thickness, with the onset of these changes seemingly differing from one quadrant to another over the course of the disease.

This study provides essential insights into the application of OCTA for predicting the development of DR in T1D children and adolescents, a context that may diverge from observations in adults with type 2 diabetes (T2D). Prior research tended to conclude that OCTA-derived retinal microvascular abnormalities were evident in the earlier stages of DR in T2D patients. For instance, Yoon et al. reported that the decreasing slope of parafoveal VSD in the DCP was not significant until the severe NPDR stage in T1D, whereas in T2D, deterioration of VSD could be found even in the early stage of DR [23]. A meta-analysis also indicated that diabetic patients without DR showed an enlarged FAZ area and reduced VAD in both the SCP and the DCP compared to healthy controls, whereas most of these differences became insignificant in T1D [24]. In contrast, our results suggested that early abnormalities in the retinal blood flow can be observed in T1D children and adolescents before the onset of DR. By employing a generalized mixed-effect model on the longitudinal data, we improved the efficiency of identifying risk factors, particularly given the challenges of handling an unbalanced dataset with limited patient numbers. Our investigation yielded valuable results after adjusting for factors influencing OCTA metrics (Fig. 2c,d; Supplementary Table 2). For example, we identified multiple DCP metrics as prognostic factors for DR, consistent with prior research emphasizing the association between DCP metrics and DR progression [25, 26]. This implies that early disturbances within the retinal microenvironment may affect DCP earlier than other regions. Moreover, our findings revealed that higher VSD in different areas and layers was linked to a lower risk of DR. This observation can be elucidated by the heightened sensitivity of VSD in capturing perfusion changes at the capillary level compared to VAD [14]. Taken together, these results not only corroborate existing evidence but also extend our knowledge of relationships between retinal blood flow and DR in children and adolescents with T1D.

It is also worth noting the comparison of the performance between peripapillary and parafoveal OCTA metrics in the clinical assessment of pediatric patients. Our findings revealed that peripapillary metrics outperformed parafoveal parameters in discriminating young eyes with NPDR, as indicated by Fig. 2c and d. Although previous studies have predominantly centered on the diagnostic utility of peripapillary OCTA in the context of glaucoma [27, 28], there is a growing body of research focusing on the optic disc vascular network in diabetic populations [29]. Unlike the macula, where blood supply is mainly attributed to smaller-diameter capillaries, the overall perfusion of the disc region is considerably influenced by larger-diameter vessels, primarily venules [30]. Therefore, our result presents an opportunity to position peripapillary metrics as a valuable predictor of abnormalities in large-vessel perfusion that can manifest at the very early stages of NPDR. However, it is imperative to approach the interpretation of these results with caution. The smaller sample size for peripapillary OCTA data analysis raises concerns about more potential for overfitting than the parafoveal dataset. Whether large-vessel perfusion dysfunction occurs earlier than previously considered remains undecided, pending validation through functional experiments.

Moreover, our findings reveal a remarkable association between DCP OCTA metrics and BCVA, suggesting that the identification of perfusion deficits in the parafoveal DCP holds clinical value for predicting vision loss in children and adolescents with T1D (Supplementary Table 4). Diabetic macular ischemia, defined as enlargement or irregularity of the FAZ and capillary dropout in the parafoveal area, is also a common comorbidity in diabetic patients. Both DR and diabetic macular ischemia can adversely affect visual function. It is generally postulated that the length of the photoreceptor layer disruption, disorganization of the retinal inner layers, and the FAZ circularity are independent determinants of visual acuity [19]. Consistent with previous research, our findings suggest that in diabetic eyes, visual impairment is more closely associated with alterations in the DCP than with the SCP [31, 32]. Decreased vessel density in the DCP is sufficient to cause visual impairment by affecting blood supply to the photoreceptors. Histologic evidence further supports the notion that the DCP may be more susceptible to ischemia-induced endothelial damage, which could play a pivotal role in modulating visual function in patients with T1D through neurovascular coupling. Additionally, we demonstrated positive correlations between BMI, AL and BCVA. Previous studies in healthy adults have also reported a connection between a higher BMI and visual impairment, implying potential links between hyperlipidemia and an increased risk of visual impairment [33]. For the effects of AL on visual function, mechanisms proposed to explain involve elongation of the eyeball resulting in the simultaneous stretching of the retina and a reduction in blood vessel width, ultimately leading to decreased overall blood flow [34].

Several confounding variables can introduce bias in quantitative OCTA analysis. First, age can cause divergent trends in retinal vasculature in childhood and adulthood. Despite most studies on healthy adults have concluded a decrease in retinal perfusion and vessel density with normal aging [35, 36], a recent study on 333 healthy Chinese children from 4 to 16 years old suggested that the retinal microvasculature continues to develop, resulting in increased VAD and VSD with age [37]. In our study of T1D children and adolescents, we observed a significantly decreasing trend of retinal vessel density with age based on linear models, suggesting that the deteriorating effect of prolonged hyperglycemia on retinal perfusion appears to outweigh the physiological retinal development. Additionally, factors such as pupil size, media opacities, and ocular pathologies can significantly impact OCTA image acquisition, potentially introducing bias [19]. To address these concerns, we administrated a standard pharmacological pupil dilation regimen to all subjects and meticulously examined the most recent slit-lamp results and medical records to exclude patients with these conditions. Moreover, most T1D patients adopt insulin therapy that potentially affects blood flow in the retina. Peng et al. reported that insulin-intensive treatment in diabetic patients can cause an instant reduction in VSD in the macular DCP and optic disc areas for 6 months compared to those treated with oral anti-hyperglycemic agents [38]. Nevertheless, the precise impact of long-term medication on retinal blood flow remains a subject of ongoing research, necessitating careful control of insulin use (including dose and frequency) in future large-scale investigations.

Furthermore, methods for OCTA image visualization and quantification may yield ambiguous results, potentially leading to misinterpretation. In our study, we used a MATLAB-based algorithm to generate quantitative metrics, consistent with our previous work for obtaining comparable observations [9]. MATLAB-based algorithms have been extensively used by researchers and demonstrated its accuracy and repeatability in recent years [6]. However, certain challenges persist, particularly regarding the DCP, which is often plagued with artefacts and projection of the SCP. Additionally, distinguishing the third vascular plexus ICP from the DCP can be difficult due to the layer segmentation border of current devices [39]. Caution is thus required when interpreting the effect of T1D on various capillary plexus. Of note, we did not observe any statistically meaningful associations between FAZ size and the onset of DR, which contradicts previous evidence highlighting the prognostic value of FAZ in DR [7]. Our FAZ area calculation depended on manually delineating the FAZ detected on OCTA, which may cause inaccuracies covering true alterations. While deep learning models have made significant advancements in the field of quantifying OCTA metrics, offering improved discrimination between non-perfusion and image artifacts, their development largely relies on extensive and diverse datasets for training [40]. For now, the acquisition of such datasets for OCTA, especially for the relatively small population of T1D children and adolescents, poses challenges, limiting the robustness of these models.

We acknowledge several limitations in the present study. First, the small sample size, especially the limited numbers of eyes with NPDR, restricts the generalizability of our results. Due to the COVID-19 outbreak, most participants from our *SCADE* cohort failed to attend annual visit. Therefore, the interpretation of these associations should be approached cautiously, and future replications in larger, independent cohorts are urgently needed. Second, we did not enroll sex- and age-matched healthy children as controls, resulting in inadequate knowledge of the physiological alterations in retinal vasculature during developmental stages. Third, the reproducibility of the measurements among different platforms was not tested. The variability of different OCTA devices may cause variations in the analysis when the same eye is imaged using different systems. Specifically, algorithms to detect flow in different platforms, artifact handling, and segmentation strategies can be sources of heterogeneity [41, 42]. Finally, DR can manifest as changes in the peripheral retinal vascular network at an early stage [6, 43]. The 6 × 6-mm scanning protocol we used in the study may be insufficient to detect distant peripheral vasculature and perfusion deficits. In recent years, widefield and ultrawidefield OCT systems have emerged that potentially address this technical limitation [44]. However, there is no consensus on whether these novel techniques are superior to commercially available devices in distinguishing patients at different stages of DR.

In conclusion, our results highlight the importance of monitoring OCTA metrics through repeated examinations and close follow-up in the clinical screening of retinopathy in T1D children and adolescents. A prominent decreasing trend in retinal perfusion and vessel density is most likely to serve as a meaningful biomarker of DR development. These findings provide a broader and more dynamic insight into the structural and functional alterations in the retinal vasculature, which may aid in designing effective prevention strategies to reduce the risk of DR.

## Supplementary Information

Below is the link to the electronic supplementary material.

Supplementary file1 (XLSX 52 KB)

## Data Availability

The authors confirm that the data supporting the findings of this study are available within the article and its supplementary materials.
